# Constitution Building

**Published:** 1888-04-28

**Authors:** 


### The Doctor's Pulpit.

### CONSTITUTION BUILDING.

A plan is the first requisite for a building of any importance. He is a very rough-and-ready workman who begins without that. A cowshed or even a barn may be thrown together without a plan, but not a respectable dwelling-house, much less a cathedral. The human constitution builder also must have a clearly-drawn plan and a definite purpose in his mind if anything strong and beautiful is to be the final result. Such a plan should include, at any rate, three main features—physical strength, intelligence, and goodness. Each of these is an indispensable part of the complete human being, and each is necessary to the other. However healthy a child may be at birth, and however physically strong he may grow up, if he be not both intelligent and virtuous the chances are that he will not carry his perfect constitution with him throughout the whole of life, but that some breach will be made which intelligence and virtue would have surely protected him against.

But the builder must know the times and seasons for his different operations. He must not put on his roof before he has built his walls, otherwise a very curious catastrophe may be the consequence. Everything must be in its own order. It seems to the present writer that the natural order is this: First, physical health; then natural intelligence; then moral goodness. Many ladies, especially unmarried governesses of uncertain age, appear to think that moral goodness should come as naturally to a child as the love of sweets, or the tendency to tumble when learning to walk. If the infant of two grabs at a piece of cake, and thereby upsets the teacup, he is a "naughty little thing;" if, in a fit of rage he seizes the unfortunate lady by the hair, and if that same hair should happen to show an unnaturally feeble attachment to the head, and come off in the little creature's hands, he is a "prodigy of wickedness," a "dreadful child," to which some untimely end is sure to come before he reaches the adolescent period. Well! well! There is a proverb in Scotland which tells us that "Auld maid's bairns have aye fine manners"; the obvious interpretation being that if those same old maids were wives and mothers they would show the present namby-pamby matrons how exactly and thoroughly Master Mischief should be taught to behave on every possible occasion. Perhaps they would; at any rate, they think they would, and that satisfies them without doing anybody else any harm. For our part, as we have already said, the development of the moral faculties should come after the opening of the intelligence—not before; and the intelligence itself should in babyhood play second fiddle to the bodily well-being. A baby in its first year is certainly not a responsible creature, and must not be treated as if it were.

Here is the newly-born infant, rosy skinned, chubby, without eyelashes; but not blind, like a kitten or a puppy. It can both scream and kick, proving at once that its muscles and lungs are in a forward state of development. What does it require? Good air, clothing, and food. A large nursery, night and day, with not too many people in it; dry, sunny, free from draughts, but with the window open, and bright cheerful walls and ceiling. As soon and as often as the weather permits, the newly-arrived one must be taken out into the open air. It cannot be out too often or too long when the weather is suitable.

As to clothing, the present writer has lived a life of continual protest against the prevalent methods of nurses. He has often wished for the determined energy of the prophet Elijah to enable him to put an end to the whole thing. But what can a medical man do against a nurse; and particularly a monthly nurse? Has she not nursed babies before the doctor was born? Has she not bandaged every one of them tightly round the stomach and the lower part of the chest? Has she not swathed them in stays and wrappages, and covered them over with mile-long and low-necked night-gowns for half a century; and is this upstart doctor to be allowed to teach his grandmother? What does he know about babies? Softly nurse! The doctor does not doubt your infallibility for a moment any more than he doubts the milkman's honesty or the butcher's power of cutting a sirloin which shall weigh exactly seven pounds. He merely suggests that if he were the baby he would like you to put him into one single loose gown of soft flannel, fitting comfortably round the neck, and not too long. He could then kick and wriggle at his pleasure; and if you were to be really so good as to gratify this baby-wish he would promise never to kick you if he could help it, and never to scream except when the soap got into his eyes or a longer pin than usual tried to find its way into the deeper parts of his anatomy. The clothing of an infant should cover every part of the body except the face and head, and should be as loose as it can well be made. Its texture should always be of soft woollen. So clothed the baby's skin can act universally and uniformly, and the susceptible internal organs—the lungs, the liver, the stomach, and the bowels are likely to be kept free from chills.

The baby's food is usually considered the most important part of baby-economy. It is at any rate a department whose importance it is impossible to exaggerate. What a fortunate thing it is that in these circumstances despised Nature promptly comes to our aid. In our wonderful times Nature is no more thought of than if she were a stupid and incompetent schoolboy. It is positively astonishing that the world has reached its present not inconsiderable stage of practical advancement without the aid of the clever people of the nineteenth century. However, here we are, and some of us are prepared to affirm that when Nature invented mother's milk she did a fairly successful stroke of business. The best thing for building the foundations of human health is mother's milk. Not, perhaps, always the milk of the infant's own mother. Sometimes that may be little better than poison. But milk from some mother, and, wherever possible, from a human mother whose baby is of the same age, or nearly, as the infant to be fed. Failing the human mother, the cow, or the goat, or the she-ass makes a not inefficient substitute. Fresh milk from a human mother or some other vertebrate female is usually to be preferred to prepared milk of any kind. Usually, but not always. In each separate case Dr Physic and Dr. Experience must hold a consultation, and decide the question at the time upon its merits.

A healthily born, intelligently clothed, and judiciously fed baby is, even as an object of virtu, much to be preferred to an oil painting which hangs on the wall. The baby is decidedly prettier, and, besides, it can multiply itself fiftyfold by its varying moods, but the picture is always the same. The baby, too, grows bigger and stronger, and has the promise of making something in after life. Who knows what he may become? In the meantime we are in no haste to get him out of his babyhood. He is a fine, healthy, happy little fellow, and fills the house with his bright and innocent laughter. Let it be the business of his mother and father to devote to the building of his constitution so much care and intelligence that when he is grown up he may be both fair to look upon, clever, and good.

